# Organizational Readiness to Implement the Chronic Disease Self-Management Program in Dialysis Facilities

**DOI:** 10.3390/geriatrics3020031

**Published:** 2018-06-14

**Authors:** Tiffany R. Washington, Tandrea S. Hilliard, Chivon A. Mingo, Rasheeda K. Hall, Matthew Lee Smith, Janice I. Lea

**Affiliations:** 1 University of Georgia, School of Social Work, Athens, GA 30602, USA; 2 American Institutes for Research, Chapel Hill, NC 27517, USA; thilliard@air.org; 3 Gerontology Institute, Georgia State University, Atlanta, GA 30302, USA; cmingo2@gsu.edu; 4 Duke University, School of Medicine, Durham, NC 27710, USA; rasheeda.stephens@duke.edu; 5 Center for Population Health and Aging, Texas A&M, College Station, TX 77843, USA; matthew.smith@tamhsc.edu; 6 School of Public Health, Texas A&M, College Station, TX 77843, USA; 7 College of Public Health, The University of Georgia, Athens, GA 30602, USA; 8 Emory University, School of Medicine, Atlanta, GA 30322, USA; jlea@emory.edu

**Keywords:** chronic kidney disease, self-management, implementation science

## Abstract

A gap exists between the development and uptake of evidence-based health promotion programs in health care settings. One reason for this gap is lack of attention to organizational readiness. The objective of this study was to assess organizational readiness to implement the Chronic Disease Self-Management Program in dialysis facilities. Survey data were collected from dialysis staff using a semi-structured Organizational Readiness for Implementing Change questionnaire. Change efficacy and change commitment scale ratings were all above 3.0, indicating a moderate level of readiness among staff. Profession and level of education were significantly associated with mean change efficacy scale ratings. Textual data revealed benefits to patients, implementation barriers and facilitators, and the influence of facility environment and culture. The findings of the current study suggest that additional efforts to advance the implementation of evidence-based health promotion programs in dialysis facilities are needed.

## 1. Introduction

A gap exists between the development and uptake of innovations—such as evidence-based health promotion programs (EBHPs)—in health care settings. Less than 15% of programs are translated into routine practice [1]. One reason for translation barriers is lack of attention to organizational readiness in settings where older adults undergo care for chronic conditions. Organizational readiness represents organizational members’ shared commitment to innovation and organizational efficacy (i.e., a collective belief to successfully implement an innovation). One-half of all implementation efforts fail because of lack of attention to organizational readiness [2,3]. This oversight is alarming because an assessment of organizational readiness determines the extent to which potential adopters view the innovation as a good fit with organizational norms and values [4]. Furthermore, such assessments could identify barriers and facilitators to EBHPs such as the well-known Chronic Disease Self-Management Program (CDSMP).

Organizational readiness for innovation assessment is important because such assessments increase implementation effectiveness by identifying organizational factors that promote or impede successful implementation [3,5]. Implementation effectiveness is facilitated by management support, availability of resources, policies and practices, values fit, and overall implementation climate [6,7]—all factors elucidated when innovation readiness assessments occur. In addition, values such as positive relationships and effective communication between organizational members may improve implementation outcomes [8].

To better inform the implementation of EBHP-related innovations (i.e., new practices, policies, or programs), the objective of this study was to assess organizational readiness for EBHPs in dialysis facilities. There are over 7000 Medicare-registered dialysis facilities in the U.S. and its territories [9]. Dialysis facilities provide individuals living with end-stage renal disease (ESRD), the irreversible stage of chronic kidney disease, life-sustaining treatment overseen by an interdisciplinary health care staff (i.e., nurses, social workers, patient care technicians, dietitians, and physicians). We were interested in examining dialysis facility staff perspectives’ on the readiness to implement a specific EBHP that promotes healthy aging, the CDSMP, because of its wide scale implementation and potential to improve health outcomes among older adults undergoing dialysis [10,11,12,13,14]. Developed at Stanford University, the CDSMP is a peer-led intervention intended to assist individuals living with chronic conditions to learn skills to manage symptoms related to their disease, and goal setting activities to improve confidence for disease self-management. CDSMP workshops traditionally consist of six sessions hosted once per week for six consecutive weeks. Each session lasts approximately 2.5 h [15]. With decades of documented success in diverse delivery sites [16], CDSMP has been shown to significantly improve participants’ physical and mental health, especially among older adults [17,18,19]. Furthermore, CDSMP has been linked to health care savings by reducing hospitalizations and emergency room visits [19].

Implementation of the CDSMP in dialysis facilities is an important endeavor given the growing prevalence of end-stage renal disease (ESRD) in the United States [20], especially among older adults who represent the fastest growing segment of the ESRD population [21]. ESRD prevalence is highest among individuals aged 65 and older, with those aged 75 and older experiencing the highest incidence rates [20]. For optimal health, individuals undergoing dialysis require adherence with a complex treatment plan involving dialysis itself, as well as prescription medications, diet and fluid restrictions, and a healthy lifestyle. Nearly 50% of individuals on dialysis experience nonadherence with their treatment plans [22], and barriers to adherence include socioeconomic status, psychological illness, and functional impairment [23,24,25,26]. These barriers may be lessened by CDSMP participation; however, the standard workflow and restricted resources available in the dialysis care setting may pose a challenge to implementation of CDSMP by dialysis facility staff. Therefore, towards a long-term goal of sustainable implementation of CDSMP in dialysis settings, we set out to examine barriers and facilitators to CDSMP implementation by dialysis facilities, as well as readiness for change factors among dialysis facility staff.

## 2. Materials and Methods

### 2.1. Theory

The current study was guided by Weiner’s organizational readiness for change theory (ORC) [3,27] and the complex innovation implementation framework (CIIF) [6]. ORC theory’s main constructs, change commitment and change efficacy, represent organizational members’ collective decision to implement change, and the extent to which they view themselves as efficacious to carry out the change [3]. ORC theory contends change is a collective effort that requires a high level of commitment from members of an organization. The CIIF is predictive of successful innovation implementation. According to the CIIF, implementation occurs after a collective decision to embed an innovation within its existing infrastructure [6,28]. Key factors that influence effective innovation implementation in health care settings include [6]: (1) management support (innovation as a management priority); (2) resource availability (resources are made available for implementation); (3) implementation policies and practices (organizational policies to facilitate implementation); (4) innovations-values fit (fit between the innovation and organization); and (5) implementation climate (innovation as an organizational priority). Taken together, ORC theory and the CIIF frame the current study’s methodology by informing the instrument selection and verbiage of open-ended questions and prompts. Of note, the authors’ intent was to understand dialysis organizational factors that facilitate or impede CDSMP implementation, as well as dialysis organizational climate and staff opinions about adoption of the CDSMP.

### 2.2. Data Collection

Data for this study were collected from staff within three independently-managed outpatient dialysis facilities of an academic medical center in a major metropolitan area in the southeastern United States. There are over 25,000 individuals living with ESRD residing in the state within which this study occurred [20]. At the time of data collection, the facilities served a predominantly Black/African American (greater than 90%) aged 55 and older patient population. Staff were notified of the study by flyers and email announcements from facility managers. Direct care and administrative staff meeting eligibility criteria (i.e., employed with the facility for a minimum of three months and English-speaking) were invited to participate in the study. Interested staff provided their names and email addresses to the facility manager, which was forwarded to the principal investigator.

Surveys were constructed in Qualtrics [29]. Staff participants received a survey link by email. The body of the email provided a description of the study. Upon opening the surveys, participants were prompted to read the consent form and provide electronic consent using their initials in order to proceed. The surveys were preceded by a two-minute video recording of the research team introducing the CDSMP’s components and benefits. The video concluded with the following prompt: “The program is typically found in community settings such as senior centers, churches, libraries, and even hospitals. I would like to bring the program to your facility because of the potential to improve health outcomes in dialysis patients. But first, I need to understand your facility’s readiness to implement the CDSMP. The questions in this survey will help me understand factors that may help or hinder implementing this program within your facility.”

The surveys were distributed to facility staff during a seven-month period (i.e., facility one May–June; facility two July–August; facility three November–December). Staff participants received a $20 store gift card for participating in the study. The study was approved by the Emory University Institutional Review Board.

### 2.3. Measures

Basic demographic questions included age, race/ethnicity, profession, and education. In addition, participations were asked to report the average number of hours worked per week, the length of time working at the current dialysis facility (in months), and the length of time working at any dialysis facility (in months).

ORC theory was operationalized using the 13-item Organizational Readiness for Implementing Change (ORIC) survey [27]. Items used a five-point Likert scale ranging from 1 (disagree) to 5 (agree). Its subscales include the seven-item Change Efficacy Scale and the five-item Change Commitment Scale. One global rating question (i.e., How ready is your facility to implement this program?) used a five-point Likert readiness scale ranging from 1 (not at all ready) to 5 (very ready). Alpha coefficients for the seven-item Change Efficacy and five-item Change Commitment Scales were 0.96 and 0.95, respectively, indicating strong inter-item consistency.

CIIF’s five constructs informed the verbiage of open-ended questions (including “please explain your answer” or “provide specific examples” where appropriate). Because fidelity—the delivery of a program as intended by its developers—is important for Chronic Disease Self-Management Program (CDSMP) effectiveness [30], an additional “fidelity readiness” construct and corresponding open-ended question was included in the survey. Constructs and corresponding questions are listed in Table 1.

### 2.4. Data Analysis

Descriptive statistical analyses were performed to summarize demographic characteristics of respondents and examine the frequency, mean, and standard deviation for each ORIC item. Alpha coefficients were computed for the seven-item Change Efficacy Scale and five-item Change Commitment Scale to assess inter-item consistency. Bivariate analyses (i.e., Spearman correlations, one-way analysis of the variance F-tests with post-hoc pairwise comparisons using the Tukey method for multiplicity) were conducted to explore associations between ORIC and global readiness ratings, and demographic characteristics; statistically significant findings are reported. All quantitative analyses were performed using Stata version 13.0 [31].

For elaboration purposes, textual data derived from the open-ended survey questions were analyzed using steps of thematic analysis [32] and guided by previous research [33]. First, the first author read the data entirely. Then, 10 percent (*n* = 6) of the participants were randomly selected using the Microsoft Excel [34] random number generator for initial open coding of the open-ended responses by the first and third authors, both of whom have conducted research involving the CDMSP [13,35] and have volunteered as CDSMP lay leaders. Next, the first and third authors generated a preliminary list of codes, discussed them, eliminated redundant codes or those irrelevant to the study’s aim [33] and collated the codes into themes. Fourth, two social work graduate students with health and gerontology interests independently coded the remaining open-ended responses using the preliminary codes as a guide. Finally, the full list of codes were collated and reconciled by the first and third authors [32].

## 3. Results

### 3.1. Quantitative

Of the 70 surveys that were emailed, 68 participants initiated surveys, and 63 were completed. The majority of respondents were Black/African American (79%); 39% were Patient Care Technicians; 32% were Nurses; 40% had completed “some college” or an Associate’s degree, and 35% had a Bachelor’s or Graduate degree (Table 2). On average, respondents were 43.8 years of age (SD = 9.2); worked 40.3 h per week (SD = 5.5); have worked at their current dialysis facility for 41.5 months (or 3.46 years; SD = 23.8); and have 111.1 months of experience working at any dialysis facility (or 9.25 years; SD = 76.0).

As shown in Table 3, mean change efficacy and change commitment item and scale ratings were all above 3.0, indicating a positive inclination toward dialysis facility organizational readiness to implement the CDSMP. Over 65% of respondents agreed or somewhat agreed with all efficacy and commitment scale items (range 66.1% to 80%). However, less than half (44%) agreed or somewhat agreed that overall, their facility was ready to implement the CDSMP (i.e., the global rating).

The global readiness rating was not associated with any dialysis facility professional demographic characteristics (i.e., profession (*p* = 0.12); education (*p* = 0.16); race (*p* = 0.06); age (*p* = 0.14); average hours worked (*p* = 0.54); length of time worked at current dialysis facility (*p* = 0.19); and length of time worked at any dialysis facility (*p* = 0.93)). However, ANOVA F tests found that profession (*p* = 0.002) and level of education (*p* = 0.007) were significantly associated with mean change efficacy scale ratings. Mean change efficacy scale ratings were higher among patient care technicians (Mean = 4.37 ± 0.33) and nurses (Mean = 4.05 ± 0.32), than social workers (Mean = 2.64 ± 1.38), dietitians (Mean = 3.74 ± 0.81), and other professionals (Mean = 3.77 ± 1.12; Table 4). Average change efficacy scale ratings were also highest among respondents who had completed technical or trade school (Mean = 4.32 ± 0.43) and some college or associates degree (Mean = 4.29 ± 0.32), compared to those who had completed high school or a GED (Mean = 3.98 ± 0.88), a bachelor’s degree (Mean = 3.86 ± 0.67), or a graduate degree (Mean = 3.10 ± 0.80). Age was moderately positively correlated with change efficacy scale ratings (Spearman’s rho (ρ) = 0.44; *p* = 0.001), indicating that higher change efficacy scale ratings were associated with increased respondent age. The relationship between change efficacy scores and average numbers of hours worked per week was not statistically significantly (Spearman’s rho (ρ) = 0.13; *p* = 0.30).

Mean change commitment scale ratings also differed significantly by profession (*p* = 0.011) and were highest among patient care technicians (Mean = 4.31 ± 0.37) and nurses (Mean = 4.10 ± 0.35) compared to social workers (Mean = 2.80 ± 1.37), dietitians (Mean = 3.6 ± 0.93), and other professionals (Mean = 3.71 ± 1.18). Age was moderately positively correlated with change commitment scale ratings (Spearman’s rho (ρ) = 0.4422; *p* = 0.0006), indicating that higher ratings are associated with increased respondent age (see Table 5).

### 3.2. Textual Data

Analysis of the textual data resulted in four themes: (1) benefits to patients; (2) implementation barriers and facilitators; (3) supportive environment; and (4) facility culture.

#### 3.2.1. Benefits to Patients

Respondents overwhelmingly viewed the CDSMP as beneficial to patients. For instance, one facility staff member said, “I think it’s an excellent program and opportunity for dialysis patients. I could [see] many patients in this clinic benefiting.” Staff cited multiple ways the CDSMP could benefit patients including promotion of self-management, peer-to-peer support, and increased quality of life. For instance, one staff member stated, “I think it will help to improve patient outlook and overall the patients’ quality of life.” Staff particularly appreciated the peer-to-peer support: “I like the fact that the patients interact [and] can look to each other for solutions. When they see someone else respond to mental and physical activity [it] gives [them] encouragement.”

#### 3.2.2. Implementation Barriers and Facilitators

While many respondents saw the benefits of the CDSMP for patients, concerns around implementation barriers were noted. For instance, one staff member spoke to limited resources to support such programs: “Usually no new resources are able to be tapped into to aid in the implementation of a new program or that program’s efficiency.” Others spoke to elements of the CDSMP’s rigid delivery criteria as a barrier to successful implementation. For instance, one staff member noted, “I question whether or not patients would be okay with sitting for another 2.5 h after already treating for 3–4 h.” However, some staff members indicated specific times it could be offer to facilitate its implementation such as “Saturday afternoons” or early in the morning “at 8:00 a.m.” One staff member spoke to the “need to do a trial run to see where it best fits”. Other implementation facilitators include staff training, transportation to increase patient participation, or adapting the program for the setting (i.e., “What would help is if the length of the session is shortened by the hours and what would help deliver this program is training classes”).

#### 3.2.3. Supportive Environment

Respondents described management and staff support as an aspect of readiness. One staff member stated, “Staff members are always looking for new ways to help achieve greater quality of life and better patient outcome. New programs are always received well by staff.” Another respondent indicated, “our manager is very good at initiating new programs”. However, at least two staff members had mixed opinions: “I don’t think there is enough support from management to implement new programs management has not been successful in the past at getting staff excited about any programs.”; and “Management would definitely be motivated to have the program but not supportive enough keep it going.” Still, staff members described their environment as highly supportive overall. When asked about resources provided by the management to support the implementation of new innovations, staff members named funds, space, materials, and personnel.

#### 3.2.4. Facility Culture

Staff offered descriptions of how innovation-related decisions were made in relation to facility culture. For instance, one staff member noted, “Any new ideas [are] always brought to the patients in writing and their opinion is always brought into consideration.” Of note, one staff member provided a rationale for why patients are included in the decision-making process: “Patients are the reasons why we all work in any facility, and if any new programs are to be implemented, they should not be kept in the dark.” Some staff members identified persons in professional roles, who, in the past, motivate buy-in for new programs: “We have social workers who can mobilize staff to help implement the program.” Overall, the facility culture was described as “always forward thinking” and consisting of “policies that support implementation of new programs”. Also, it was clear that decisions about implementing new programs were prioritized when they were in the best interest of the patients as reflected in the following two quotes: “We want patients to take responsibility for their own health care decisions and be an integral part of their health care team and I think this program will help them achieve this.”; and “The CDSMP fits into our mission by seeking to improve patient outcomes.”

## 4. Discussion

ESRD is a complex condition that undermines quality of life in older adults [36]. The CDSMP could facilitate quality of life improvements, but there is limited information about organizational readiness to implement it in dialysis facilities where older adults undergo ESRD treatment. This study was conceived to serve as a precursor to a pilot test of the CDSMP within dialysis facilities. To the authors’ knowledge, less than 1% of all CDSMP implementation has occurred using the built environment of a dialysis facility [11,37,38,39]. As suggested in other chronic disease-focused intervention research, embedding such programs within the built environment of a health care facility as unique as a dialysis facility may promote sustainability and translation [35,40,41]. Moreover, the delivery site matters for CDSMP completion rates. For instance, Black/African American individuals prefer health care organizations over senior centers and other settings [42]. Still, there have been few wide-scale implementation attempts of a comprehensive, evidence-based health promotion intervention in dialysis facilities, and yet there is a great need to do so considering dialysis patients in a previous study evidenced low self-management in exercise (range 0–20, mean 2.5), patient–provider communication (range 0–15, mean 2.5), and cognitive symptom management (range 0–30, mean 0.9) [14]. Furthermore, given that patients spend an average of 12 to 15 h per week in dialysis facilities suggests the built environment may promote its sustainability. Finally, 78% of dialysis patients affirmed they would participate in a self-management program if it were offered in their facility [14]. This finding is not surprising given that a previous attempt to offer the CDSMP to dialysis patients resulted in a 74% mean attendance rate [43], and modifications to the delivery format could facilitate participation [44].

The findings of the current study point to moderate readiness for the implementation of the CDSMP in a dialysis facility. Mean ratings for the change efficacy and change commitment scales were above 3.0, indicating a positive inclination toward dialysis facility organizational readiness to implement the CDSMP (although, to establish the exact thresholds that indicate low, moderate, or high change will require future research). Of all ORIC items, the majority of respondents (80%) agreed or somewhat agreed the facility organization can support staff as they adjust to CDMSP implementation. However, over half (56%) had low levels of agreement with readiness to implement the program, suggesting that although most feel self-efficacious and committed to implementing the CDSMP, potential barriers exist. Fortunately, the textual data identified those barriers: limited resources to support implementation and the CDMSP’s rigid delivery. These same barriers were identified in previous research examining factors that support CDSMP’s implementation [45]. Other barriers include unsupportive leadership, limited training, and lack of demand for the program [46].

Interestingly, the “front-line” dialysis staff, patient care technicians and nurses, had higher change efficacy scale ratings than social workers and dietitians. This finding could be related to differentiating roles: patient care technicians and nurses work collaboratively to oversee the dialysis treatment procedures and in-center medication management, whereas social workers and dietitians are removed from the “on-the-floor” technical aspects of dialysis care. Therefore, questions that relate to “handling the challenges that might arise” and “coordinating tasks” may have meaning of a more clinical nature for the former. However, this speculation would require further exploration because the differences may also relate to the smaller number of social work and dietitian respondents (*n* = 6 and *n* = 5, respectively) in comparison to patient care technician and nurse respondents (*n* = 24 and *n* = 20, respectively). Furthermore, because patient care technicians and nurses represented the majority of respondents, a future study may focus solely on dialysis organization leaderships’ (e.g., administrators, medical directors) influence on and perspectives about change readiness.

### 4.1. Implications

The current study is a contribution to social and behavioral sciences research because limited literature exists that provides guidance on the implementation and dissemination of evidence-based interventions in a unique health care setting (e.g., dialysis facilities). In fact, widespread dissemination and implementation of EBHPs has proven to be a challenge for researchers and community stakeholders, thus, creating a research-to-practice gap that has public health implications [1]. However, our research begins to identify barriers and facilitators to the diffusion of EBHPs within dialysis facilities by considering the impact of readiness. Although the complexities around dissemination and implementation are many, realizing the importance of assessing readiness in preparation for widespread implementation efforts is important because behavioral intervention research continues to experience either a lag or deficiency between effectiveness research and sustainable program dissemination and implementation [1]. Leveraging a partnership with unique or specialized health care settings is an approach that is underutilized. Our findings bring light to the benefits of assessing and predicting potential success prior to attempting implementation with this context. For example, assessing readiness for CDSMP in settings like dialysis facilities could call attention to the need for intervention adaptations. For instance, participants in this study indicated that the rigid structure for CDMSP designed by the developers may be a barrier to the adoption in traditional dialysis clinics. Therefore, the length of time to widespread adoption may be minimized by addressing this barrier in the pre-implementation phase of the research. The mention of this issue further supports the idea that context matters, and there is not a one-size-fits-all method for implementing behavioral interventions at the individual-, community-, or health care setting-level. To the authors’ knowledge, no studies have assessed organizational readiness to innovation implementation in dialysis facilities, and use of ORC theory and the CIIF allows for an organizational- rather than individual-level assessment of implementation readiness (e.g., individual skills and knowledge). It is hoped that findings from this study will advance knowledge for effective methods to integrate evidence-based disease self-management interventions in dialysis settings.

Indeed, there are many other reasons why innovations are slow to spread in health care facilities including high costs, low-quality implementation studies, and an inadequate understanding of benefits and risks associated with the adoption of an innovation [1,47,48]. The published guidance on designing and developing new programs exceeds the published guidance on implementing and sustaining new programs [1]. In addition, implementation efforts are rarely guided by theory- and empirically-informed implementation frameworks [6]. Such frameworks could delineate organizational features that may improve implementation effectiveness and increase the likelihood of sustainability. Also, individuals undergoing dialysis and dialysis facility staff may experience barriers to participating in research studies that serve as early tests of EBHPs. For example, patients may have a limited understanding of research or competing personal priorities to engage in research (e.g., time constraints, transportation limitations), and staff may have inadequate research expertise [48]. Finally, failure to monitor fidelity, the extent to which an intervention is delivered as intended, impedes successful implementation and long-term sustainability [49]. These and other reasons—such as poorly conceptualized study designs of interventions [50] and failure to delineate the core components of social or behavioral interventions that would allow potential adopters to make decisions about adaptability—will continue to widen the research-to-practice gap.

### 4.2. Limitations

This study’s findings are not generalizable due to small, non-probabilistic sample. Tests of ORC theory typically requires multi-site health care organizations; this study captures perspectives from 63 staff members across three interconnected facilities, only. Furthermore, it is not clear how factors such as workload, burnout, and turnover relates to organizational change readiness. It seems important to understand these relationships because one in three dialysis nurses experience burnout primarily due to workload [51] and caseloads of full-time dialysis social workers average 116 patients [52]. Finally, our participation rate was high because only respondents expressing interest in the study were selected to complete the survey. Nevertheless, the current study provides insight into organizational factors that relate to the implementation of EBHPs in dialysis facilities—an area that has received little attention thus far.

## 5. Conclusions

Health care facilities are the second largest delivery site of the CDSMP nationwide [10]. An examination of facility readiness to implement evidence-based programs is one way to improve the uptake of such programs into actual care settings. Such an examination may provide insight on how to implement EBHPs, and inform the research design of interventions aimed to increase uptake. Dialysis facilities, in particular, are ripe for implementation of EBHPs. Perhaps the best quote from this study that reflects this claim is:

I believe providing quality care goes beyond dialysis treatments. It is about looking at a patient as a whole person, with physical and emotional ups and downs like anyone else. Dialysis has a huge impact on patient’s quality of life and livelihood. I believe it’s crucial for mental and physical help to join support programs. This program would be particularly beneficial to patients as they will have an outlet, and mentors who can relate and provide a realistic perspective or guidance.

## Figures and Tables

**Table 1:** Operational definitions

Construct	Example Questions (Staff)
Management Support	Does the facility’s management support the implementation of new programs like the Chronic Disease Self-Management Program?
Resource Availability	What resources are made available when the facility implements a new program?
Implementation Policies and Practices	Do the facility’s policies support the use of the Chronic Disease Self-Management Program? Please describe.
Innovation Values-Fit	Describe ways the Chronic Disease Self-Management Program fits with the dialysis facility’s mission to provide quality care.
Implementation Climate	Describe the staff’s attitude toward introducing an evidence-based intervention such as the Chronic Disease Self-Management Program.
Fidelity Readiness	What helps staff members deliver programs as they were intended? What are barriers to delivering programs as they were intended?

**Table 2:** Descriptive statistics.

Respondent Characteristics (*N* = 63) ^a^	N	%	Mean	*SD*	Min	Max
Age	57		43.8	9.2	25.0	63.0
Race/Ethnicity	62					
White	3	4.8				
Black	49	79.0				
Asian/Pacific Islander	5	8.1				
Other_Specify ^b^	5	8.1				
Profession	62					
Nurse	20	32.3				
Social Worker	6	9.7				
Dietitian	5	8.1				
Patient Care Technician	24	38.7				
Other_Specify ^c^	7	11.3				
Education	63					
Completed High School or GED	6	9.5				
Technical or Trade School	10	15.9				
Some college or Associate’s degree	25	39.7				
Bachelor’s Degree	11	17.5				
Graduate Degree	11	17.5				
Average Number of Hours Worked Per Week	63		40.3	5.5	16.0	66.0
Length of Time Working at Current Dialysis Facility (in Months)	62		41.5	23.8	3.0	120.0
Length of Time Working at Any Dialysis Facility (in Months)	62		111.1	76.0	3.0	336.0

^a^ 70 surveys that were emailed, 68 participants initiated surveys, and 63 were completed. ^b^ Other Specified: 1—Asian/White, 2—Black/Blackfoot Indian, 1—Garifuna, 1—Brown Tone. ^c^ Other Specified: 3—Administrative Assistant, 2—Financial Counselor, 1—Lead Lab Tech, 1-Unspecified.

**Table 3:** Perceptions of dialysis facility organization-level change efficacy and change commitment.

Variable	N	Mean ^a^	SD	Scale Response ^b^	
				Agree/Somewhat Agree %	Disagree/Somewhat Disagree/Neither Agree Nor Disagree %
**Change Efficacy Item Ratings**					
We can get staff invested in implementing this program.	62	3.92	1.12	74.2	25.8
We can keep track of progress in implementing this program.	60	4.07	0.97	80.0	20.0
We can support staff as they adjust to this program.	60	4.07	1.06	80.0	20.0
We can keep the momentum going in implementing this program.	59	3.95	1.11	71.2	28.8
We can handle the challenges that might arise in implementing this program.	60	3.90	1.10	73.3	26.7
We can coordinate tasks so that implementation goes smoothly.	60	4.00	1.07	71.7	28.3
We can manage the politics of implementing this program.	60	3.73	1.06	66.7	33.3
Change Efficacy Scale Rating.	62	3.98	0.96		
**Change Commitment Item Ratings**					
We are committed to implementing this program.	62	3.97	1.06	71.0	29.0
We will do whatever it takes to implement this program.	59	3.95	1.09	67.8	32.2
We want to implement this program.	60	4.00	1.07	66.7	33.3
We are determined to implement this program.	59	3.92	1.07	66.1	33.9
We are motivated to implement this program.	59	3.88	1.08	67.8	32.2
Change Commitment Scale Rating	62	3.97	0.99		
**Global Rating of Readiness**					
How ready is your facility to implement this program?	61	3.07	1.31	44.3	55.7

^a^ Change efficacy scale (seven item) and change commitment scale (five items), item mean score. ^b^ Scale responses were dichotomized in ‘agree/somewhat agree’ and ‘disagree/somewhat disagree/neither agree nor disagree’.

**Table 4:** Change efficacy ^a^.

Variable	Sig
Profession ^b^	0.002 *
Level of education ^c^	0.007 *
Age ^d^	0.001 **
Race	0.60
Average Hours	0.30
Length of Time Worked at Current Facility ^e^	0.06
Length of Time Worked at Any Facility ^e^	0.07

^a^ In dialysis organizational climate and staff opinions about the adoption of a specific innovation. ^b^ Profession of staff was significantly associated with mean change efficacy scale ratings; * *p* < 0.05. ^c^ Level of education were significantly associated with mean change efficacy scale ratings; ** *p* = 0.001. ^d^ Moderately positively correlated with change efficacy scale; * *p* < 0.05. ^e^ In months.

**Table 5:** Change commitment ^a^.

Variable	Sig
Profession ^b^	0.011 *
Level of education	0.06
Age ^c^	0.0006 **
Race	0.11
Average Hours	0.13
Length of Time Worked at Current Facility ^d^	0.08
Length of Time Worked at Any Facility ^d^	0.41

^a^ Dialysis organization collective commitment to adopt a specific innovation. ^b^ Profession of staff was significantly associated with mean change commitment scale ratings; * *p* < 0.05. ^c^ Moderately positively correlated with change commitment scale ratings; ** *p* < 0.001. ^d^ In months.
